# Isolation and Characterization of Mouse Choroidal Melanocytes and Their Proinflammatory Characteristics

**DOI:** 10.3390/cells14090646

**Published:** 2025-04-28

**Authors:** Yong-Seok Song, SunYoung Park, Debra Fisk, Christine M. Sorenson, Nader Sheibani

**Affiliations:** 1 Department of Ophthalmology and Visual Sciences, University of Wisconsin School of Medicine and Public Health, Madison, WI 53705, USA; song224@wisc.edu (Y.-S.S.); spark67@wisc.edu (S.P.); dfisk@wisc.edu (D.F.); 2 Mcpherson Eye Research Institute, University of Wisconsin School of Medicine and Public Health, Madison, WI 53705, USA; cmsorenson@pediatrics.wisc.edu; 3 Department of Pediatrics, University of Wisconsin School of Medicine and Public Health, Madison, WI 53705, USA; 4 Department of Cell and Regenerative Biology, University of Wisconsin School of Medicine and Public Health, Madison, WI 53705, USA

**Keywords:** choroid, inflammation, age-related macular degeneration, cell adhesion molecules, integrins, VEGF receptors, c-Kit, stem cell factor

## Abstract

Melanocytes are a major cellular component of the choroid which aids in the maintenance of choroidal integrity and vision. Unfortunately, our knowledge regarding the cell autonomous melanocyte function, in preserving choroidal health and the ocular pathologies associated with choroidal dysfunction, remain largely unknown. The ability to culture melanocytes has advanced our knowledge regarding the origin and function of these cells in choroidal homeostasis and vision. However, the culture of murine choroid melanocytes has not been previously reported. Here, we describe a method for the isolation of melanocytes from the mouse choroid, as well as the delineation of many of their cellular characteristics, including the expression of various cell-specific markers, cell adhesion molecules, melanogenic capacity, and inflammatory responses to various extracellular stressors. Unraveling the molecular mechanisms that regulate melanocyte functions will advance our understanding of their role in choroidal homeostasis and how alterations in these functions impact ocular diseases that compromise vision.

## 1. Introduction

Uveal melanocytes comprise a major cellular component of the choroid in the eye. Along with choroidal myeloid cells, they populate the choroid, early in development and preceding the appearance of the vascular structures [1]. Melanocytes are neural crest-derived cells [2] that produce melanin, a pigment that scatters light and has both antioxidant [3,4] and photoprotective qualities [5,6]. In mammal skin and hair follicles, melanocytes synthesize melanin throughout the life of the cells. This process is influenced by environmental factors such as ultraviolet (UV) radiation and α-melanin-stimulating hormone (α-MSH). In contrast, non-cutaneous melanocytes found in the eye, ear, and some internal organs [7] do not usually produce melanin in response to environmental factors [8]. Later in choroid development, melanocytes appear closely associated with the vascular layers below the choriocapillaris [9]. Their presence in the choroid has recently been shown to be important for the proper vascularization and maintenance of the choroidal vasculature using melanocyte-deficient mice [1].

The role of uveal melanocytes in providing pigmentation to absorb light is well established. Their malignant transformation leads to uveal melanoma, the most common eye tumor, and the most common site of melanoma after the skin [10,11,12,13]. In addition, the pathogenesis of sympathetic ophthalmia and Vogt–Koyanagi–Harada syndrome is linked to the uveal melanocyte dysfunction. Damage to uveal melanocytes leads to eye diseases such as age-related macular degeneration (AMD), cataracts, and other inflammatory disorders [4,14,15,16]. Our knowledge regarding the physiology and pathophysiology of uveal melanocytes is facilitated by our ability to culture these cells. Thus, advances in new gene targeting strategies and culturing of uveal melanocytes will allow us to continue and better understand their significant roles in the development of the choroid and their potential dysfunction under various pathological conditions.

There have been many attempts to culture uveal melanocytes from human and non-human primates [10,17,18]. Advances in the cultivation of dermal melanocytes have led to well-delineated isolation techniques, purification, and cultivation of uveal melanocytes. Hu et al. [10] were the first to report a successful method for the isolation, purification, and culture of uveal melanocytes from adult human donor eyes. Using these methods, numerous laboratories have now reported the isolation and study of human uveal melanocytes [4,7,19,20,21,22]. To the best of our knowledge, there is no report of uveal melanocytes cultured from mouse eyes. The isolation and propagation of uveal melanocytes from wild type and transgenic mice will allow us to gain new insights into the cell autonomous regulatory mechanisms that impact various functions of uveal melanocytes, and likely the choroid.

One limitation of the cultures of uveal melanocytes is their limited life span and proliferation capacity for use in biochemical studies. This is further hampered by the small number of these cells in the rodent eyes limiting their successful isolation. Here, we have adapted our novel method of isolating ocular cells from the Immortomice to isolate choroidal melanocytes. These mice ubiquitously express a temperature-sensitive large T-antigen, which is only expressed at the permissive temperature of 33 °C. The expression of large T-antigen is driven by a low concentration of interferon-ɣ. Furthermore, the culture of these cells at 37 °C in the absence of interferon-ɣ for 48 h results in complete large T-antigen degradation. Using this method, we were first to report the successful isolation of retinal vascular cells [23,24,25] and, more recently, choroidal vascular and retinal pigment epithelial cells from wild-type and transgenic mice [26,27]. We now report the isolation and characterization of uveal melanocytes from C57BL/6J Immortomice. This method can be readily applied to Immortomice crossed with desired transgenic mice to study gene-specific effects on the phenotype and biochemical characteristics of these cells, as we have reported for other cell types from different transgenic mice [27,28].

## 2. Materials and Methods

### 2.1. Ethics Statement

The experiments shown here were performed in accordance with the Association for Research in Vision and Ophthalmology Statement for the Use of Animals in Ophthalmic and Vision Research. The protocols were approved by the Institutional Animal Care and Use Committee of the University of Wisconsin School of Medicine and Public Health (IACUC assurance number: D16-00239). Euthanasia by CO_2_ asphyxiation was carried out according to approved protocols.

### 2.2. Experimental Animals

Immortomice expressing a temperature-sensitive SV40 large T antigen were obtained from Charles River Laboratories (Wilmington, MA, USA) and back-crossed to C57BL/6J mice for more than 10 generations, as previously described [23,24]. Mice containing the immorto gene were identified by PCR analysis of DNA isolated from tail biopsies. The PCR primer sequences were as follows: Immorto Forward: 5′-CCTCTGAGCTATT CCAGAAGTAGTG-3′, Immorto reverse: 5′-TTAGAGCTTTAAATCTCTGTAG GTAG-3′.

### 2.3. Tissue Preparation and Culture of ChMC

Melanocytes were isolated from mouse choroid by collecting choroid from one litter (3-7 pups, 4-week-old) of positively identified Immortomice. Under a dissecting microscope in cold Dulbecco’s Modified Eagle’s Medium (DMEM; D5523, Sigma, St Louis, MO, USA), the anterior eye portion was removed, followed by the lens, vitreous, retina, and optic nerve, leaving only a tissue composed of RPE, choroid, and sclera. Choroidal tissue was placed in cold, serum-free, basal Ham’s F10 medium (Nutrient Mixture F10 Ham (N6635, Sigma) with 1.2 g sodium bicarbonate (BP328500; ThermoFisher Scientific, Rockford, IL, USA). The tissue was then transferred to a 60 mm dish (12556001; ThermoFisher Scientific) where it was minced and digested in a 50/50 mix of Collagenase IV (LS004186) 100 U/mL and Dispase (LS02109) 1 U/mL (Worthington, Lakewood, NJ, USA) in serum-free Ham’s F10 basal medium for 30 min at 37 °C. The digested tissues were rinsed with Ham’s F10 medium containing 10% fetal bovine serum (FBS) and centrifuged for 5 min at 500× *g*. The digested tissue pellet was again rinsed in Ham’s F10 medium containing 10% FBS, passed through a 70 µm cell strainer (352350; Falcon, ThermoFisher Scientific) and pelleted. Cells were resuspended in 2 mL Ham’s F10 medium containing 10% FBS, 2 mM l-glutamine (25030081), 1% nonessential amino acids (NEAA; 11140050), 100 μg/mL of streptomycin and 100 U/mL penicillin (15140122) (ThermoFisher Scientific), and 44 U/mL recombinant murine interferon-γ (485MI100; R&D Systems, Minneapolis, MN, USA). Cells were deposited in one well of a 24-well tissue culture plate (12556006; BioLite, ThermoFisher Scientific) and maintained at 33 °C with 5% CO_2_. Cells were progressively passed to larger plates and ultimately maintained and propagated in 60 mm dishes. Our immunofluorescence staining with melanocyte and RPE cell markers indicated that nearly all the cells in these isolations were positive for melanocyte markers but negative for keratins. Thus, additional steps were not needed to remove contaminating cells as previously reported [10]. These cells were also negative for endothelial cell marker Cd31, pericytes marker (Pdgfrb), and RPE cell marker (Rpe65) confirmed by qPCR (see Section 3).

### 2.4. Isolation and Culture of Other Cell Types

Choroidal and retinal endothelial cells (ChEC and REC) were isolated and maintained from Immortomice as described in previous papers [26] and grown in low-glucose DMEM (D5523, Sigma) containing 10% FBS, 2 mM l-glutamine, 2 mM sodium pyruvate (11360070), 20 mM HEPES (15630080) (ThermoFisher Scientific), 1% NEAA, 100 μg/mL of streptomycin and 100 U/mL penicillin, 55 U/mL heparin (H3149-125KU; Sigma), 100 μg/mL endothelial growth supplement (E2759; Sigma), and 44 U/mL recombinant murine interferon-γ (R&D Systems). Retinal pigmented epithelium (RPE) cells were isolated as described in a previous paper [27] and grown in low-glucose DMEM containing 10% FBS, 2 mM l-glutamine, 1% NEAA, 100 μg/mL of streptomycin and 100 U/mL penicillin, and 44 U/mL recombinant murine interferon-γ. ChEC, REC, and RPE cells were grown on 60 mm plates coated with 1% gelatin (G8150; Sigma) prepared in PBS (D1408; Sigma), and maintained at 33 °C with 5% CO_2_. Choroidal pericytes (ChPC) were isolated and characterized as described for retinal pericytes [25]. ChPC were grown on non-coated plates in low-glucose DMEM containing 10% FBS, 2 mM l-glutamine, 1% NEAA, 100 μg/mL of streptomycin and 100 U/mL penicillin, and 44 U/mL recombinant murine interferon-γ. Although immorto cells, by definition, can proliferate indefinitely, in the studies presented here, only cells from P3–P14 were used. From our experience with the murine immorto cells, we know during this passages period, there are minimal changes in the expression of cell-specific markers, which began to change with later cell passages after P14.

### 2.5. Morphology Evaluations

Images of newly isolated and early passage (passage 0–3) ChMC were obtained for visible morphological and pigment content comparisons. The cells were photographed in digital format on an inverted microscope (Eclipse TS100; Nikon, Tokyo, Japan) with a 12-bit camera (QICAM, QImagining, Teledyne Photometrics, Tucson, AZ, USA) and processed using QCapture Pro (Teledyne Photometrics) when they reached approximately 75–85% confluence in their respective growth plates. Higher passage ChMC, ChEC, and RPE cells were also photographed in a similar way at a similar confluence to allow for qualitative morphological comparison of the different cell types.

### 2.6. Immunostaining of ChMC

The surface of 4-well glass chamber slides (PEZG0416; Sigma) was coated with 0.5 mL of fibronectin (CB40008; ThermoFisher Scientific) at 2 μg/mL prepared in serum-free DMEM for at least 4 h. The wells were rinsed with 0.5 mL of PBS and cells (4.0 × 10^4^) in 1 mL of growth medium were added to each well and incubated overnight. The next day, cells were rinsed with 0.5 mL of PBS and fixed with 0.5 mL of 4% paraformaldehyde (PFA, 15710; Electron Microscopy Sciences, Hatfield, PA, USA) prepared in PBS on ice for 15 min. Cells were then rinsed with 0.5 mL of PBS and permeabilized with 0.5 mL of 0.1% Trixon-X100 (BP-151; ThermoFisher Scientific) prepared in PBS for 10 min at room temperature. Once fixed and permeabilized, the cells were incubated with 0.250 mL of blocking buffer (1% bovine serum albumin (BSA; BP9703, ThermoFisher Scientific) in Tris-buffered saline (TBS; 20 mM Tris-HCl, 150 mM NaCl, pH 7.6)) for 1 h at room temperature. Primary antibodies were diluted in blocking buffer 0.25 mL (1:200) and added to the cells and incubated at 4 °C overnight. The next day, cells were rinsed with TBS and incubated with fluorescein isothiocyanate (FITC)-conjugated appropriate secondary antibodies prepared in 0.25 mL of blocking buffer (1:500) for 1 h at room temperature. Cells were then rinsed with TBS (three times, 0.5 mL each) and incubated with 0.25 mL of DAPI-diluted in a blocking buffer (1:1000) for 1 min at room temperature. Cells were then rinsed with TBS, and the slide was mounted using mounting medium (0100-01; Southern Biotech, Birmingham, AL, USA). Cells were photographed on a Zeiss Axiophot fluorescence microscope (Carl Zeiss Optical, New York, NY, USA) equipped with a digital camera. Antibodies and reagents used included: Melan A antibody (PA5-99174), Pmel antibody (PA5-95605), Pan Cytokeratin antibody (MA5-12231) and DAPI (D1306I; ThemoFisher Scientific), and FITC AffiniPure goat anti-rabbit IgG antibody (111-095-045) and FITC AffiniPure donkey anti-mouse IgG antibody (715-095-151; Jackson ImmunoResearch, West Grove, PA, USA).

### 2.7. Flow Cytometry Analysis

The 60 mm culture plates containing a monolayer of ChMC, ChEC, or RPE cells were rinsed with PBS containing 0.04% EDTA (E6758, Sigma) and incubated with 1.5 mL TBS (pH 7.6) containing 2 mM EDTA and 0.05% BSA for 15 min at 37 °C. Cells were rinsed from plates with low glucose DMEM containing 10% FBS and blocked in TBS with 1% goat serum for 20 min on ice. Cells were then fixed with 2% PFA in PBS for 30 min at 4 °C. Cells were washed with TBS containing 1% BSA and incubated on a low-speed orbital shaker with primary antibodies for 30 min at 4 °C. Anti-Mitf (A1255), anti-Mlana/Melan-a (A6290), anti-Slc16a3 (A10548, ABClonal, Woburn, MA, USA), anti-Mc1r (PA575342, ThermoFisher Scientific), anti-Trp2 (Sc-74439, Santa Cruz Biotechnology, Dallas, TX, USA), anti-Pmel (PAS101023), anti-c-Kit (105817), and anti-S100β (676603; BioLegend, San Diago, CA, USA) antibodies were used to analyze the expression of melanocyte markers. Anti-Pan Cytokeratin (MA528561, ThermoFisher Scientific) antibody was used to exclude the possibility that these cells are RPE cells which are positive for cytokeratins, while melanocytes are negative. For integrin expression analysis, anti-α1 (555001), β4 (553745, BD Biosciences, Franklin Lakes, NJ, USA), α2 (AB1936), α3 (AB1920), α4 (AB1924), α5 (AB1921), α5β1 (MAB1999), β1 (MAB2000), β2 (MABT42), β3 (MAB1957, Millipore, Burlington, MA, USA), β5 (sc-5401), and β8 (sc-25714, Santa Cruz Biotechnology) were used. Anti-VEGFR-1 (MAB471), VEGFR-2 (MAB443; R&D Systems), ICAM-1 (sc-1511 clone M-19; Santa Cruz Biotechnology), ICAM-2 (553326), and VCAM-1 (559165; BD Biosciences, San Diego, CA, USA) were also used. Cells were washed twice with TBS with 1% BSA and incubated on a low-speed orbital shaker with the appropriate FITC-conjugated secondary antibody (Jackson ImmunoResearch) for 30 min at 4 °C. Cells were washed twice with TBS with 1% BSA, resuspended in 0.5 mL TBS with 1% BSA, and analyzed by an Attune NxT analysis cytometer (ThermoFisher Scientific). All antibodies were used at the recommended dilutions provided by the supplier. Cells incubated with secondary antibodies in the absence of primary antibodies were used as controls.

### 2.8. Reverse Transcription Quantitative PCR Analysis (RT-qPCR)

RNA was prepared from cells actively growing in 60 mm tissue culture plates. Total RNA was extracted using a combination of TRIzol reagent (15596026; Life Technologies, Grand Island, NY, USA) and RNeasy mini kit (74104; Qiagen, Maryland, CA, USA) column for purification. The cDNA synthesis was performed from 1 μg of total RNA using the RNA to cDNA EcoDry Premix (Double Primed) kit (Takara, San Jose, CA, USA). Ten-fold dilutions of cDNA were used as templates in qPCR assays, performed in triplicate on QuantStudio3 real-time PCR system (ThermoFisher Scientific) using the TB-Green Advantage qPCR Premix (639676; Takara). The amplification conditions used were as follows: 95 °C for 2 min; 40 cycles of amplification (95 °C for 15 s, 60 °C for 40 s); dissociation curve step (95 °C for 15 s, 60 °C for 15 s, 95 °C for 15 s). The linear regression line for nanograms of DNA was assessed from relative fluorescent units (RFUs) at a threshold fluorescence value (Ct). Expression levels of target genes were quantified by comparing the RFU at the Ct to the standard curve and normalized by simultaneous amplification of 60S ribosomal protein L13α (Rpl13a), used as a housekeeping gene. The expression of other cell-type-specific markers Cd31 (ChEC marker), Pdgfrb (ChPC marker), and Rpe65 (RPE marker) were used to demonstrate the specificity of ChMC compared with melanocyte markers, including Mc1r, Mitf, and Tryptase. We also examined the expression of proinflammatory genes known to be expressed in human ChMC under basal and stress conditions by qPCR. The list of primers is provided in Table 1.

### 2.9. Choroid/RPE Tissue Staining of Melanocytes

For the immunofluorescent staining (IF) of melanocytes, the eyeballs from 8-week-old non-pigmented FVB/NJ mice (Jackson Labs, Bar Harbor, ME, USA) were collected and fixed for one hour in 4% PFA at room temperature and then washed in PBS (3 times). The choroidoscleral complex was then dissected in PBS, and incubated in blocking solution (1% BSA, 0.3% triton X-100 and 0.05% sodium azide in PBS) for 1 h on a shaker. Choroidoscleral complex was then incubated with rabbit anti-S100β antibody (ab52642, Abcam, Waltham, MA, USA) and goat anti-Mitf antibody (AF5769-SP; R&D Systems) diluted 1/250 in the blocking buffer, and fresh antibodies were added daily for 3 days at room temperature on a shaker. Following incubation, choroidoscleral complex was washed in PBS (3 times) before their incubation with donkey anti-rabbit-Cy3 (711-165-152) and donkey anti-goat Alexa Fluor 647 (705-605-147) secondary antibodies (Jackson ImmunoResearch Labs) both diluted 1:500 in blocking buffer and incubated for 5 h at room temperature on a shaker. The choroidoscleral complex was then mounted, and images were captured using Nikon A1 confocal microscope. Images were processed using NIS-Elements Software (version 5.30.03; Build 1549).

### 2.10. Melanogenesis Studies

To assess the melanogenic capacity of ChMC in response to external stimuli, we examined the expression of genes associated with melanogenesis including *Mc1r*, *tyrosinase*, *Pmel*, and *Mitf* in cells incubated with the Mc1r agonist, BMS 470,539 (22231; Cayman Chemical Company Inc., Ann Arbor, MI, USA). ChMCs were incubated with vehicles or BMS 470,539 (1 µM) prepared fresh in a melanocyte culture medium for 72 h. Total RNA was prepared and subjected to qPCR analysis using specific primers (Table 1) as detailed above. These experiments were repeated with three different isolations of ChMC.

### 2.11. The Impact of Extracellular Stressors on Inflammatory Properties of ChMC

ChMCs are proposed to have a regulatory role in the modulation of the inflammatory processes. However, the detailed mechanisms involved remain largely elusive. To investigate the impact of stress conditions on inflammatory characteristics of ChMC, we incubated ChMC with various stressors, including sodium iodate (NaIO_3_, S4007; Sigma), ATP, and adenosine. We first evaluated the viability of ChMC following incubation with various concentrations of these stressors for 24 h using the Cell Counting Kit-8 as recommended by the supplier (Dojindo Molecular Technologies, Inc.; Rockville, MD, USA). Using concentrations with minimal cytotoxic effects, we assessed the impact of these stressors on the expression of various inflammatory mediators. Bz-ATP (an analog of ATP) and NECA (an analog of adenosine) (Sigma) were used for these studies. We also examined the expression of ATP receptor (P2X7) and adenosine receptors (Adora1, Adora2a, Adora2b, and Adora3) in ChMC by qPCR and specific primers as detailed above (Table 1). For inflammatory mediators, we examined the expression of IL-1β, IL-6, Tnf-α, Mcp-1, Nos2, Tgf-β1, and Thbs1, which are detected in mice with choroidal injury [29], and human ChMC incubated with lipopolysacchride (LPS) [30].

### 2.12. Statistical Analysis

For more than two samples, one-way ANOVA followed by Tukey’s multiple-comparison test using GraphPad Prism 8.0 (GraphPad Software, San Diego, CA, USA) was used for the statistical differences between groups. The significant differences between the means of every possible two groups in all experimental groups was assessed using Tukey’s multiple-comparison test. Student’s unpaired *t*-test (two-tailed) was used for statistical analysis between two groups. Mean ± standard error is shown. *p* < 0.05 was considered significant.

## 3. Results

### 3.1. Isolation of Mouse ChMC

**Figure 1:**
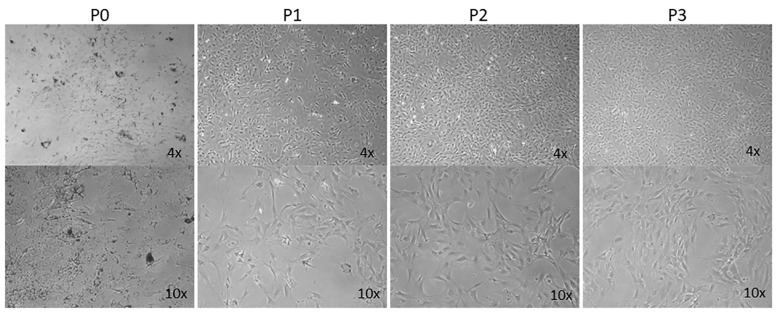
Morphology and melanization of mouse choroidal melanocytes in culture. Choroidal melanocytes from Immortomice were cultured on uncoated plates. The cells, which have a morphology like melanocytes from previous studies, were photographed shortly after isolation (P0) and after each passage up to the third passage (P1, P2, and P3, respectively) in digital format using a Nikon microscope using a 4× or 10× objective (final magnification: top panels ×40, bottom panels ×100). The number of cells containing melanin decreases with each successive passage, as has been previously observed by others culturing melanocytes.

**Figure 2:**
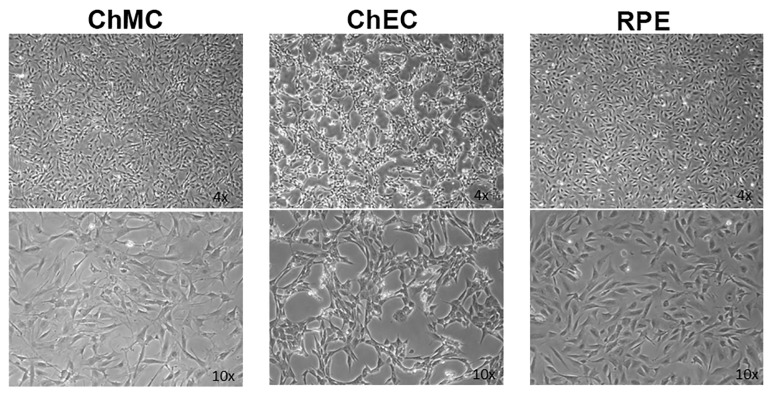
Morphological comparison of mouse choroidal melanocytes (ChMCs), choroidal endothelial cells (ChECs), and retinal pigmented epithelium (RPE) cells in culture. Actively growing early-passage (P7) cultures of ChMC, ChEC, and RPE cells were photographed using a Nikon phase microscope in digital format using a 4× or 10× objective (final magnification: top panels ×40, bottom panels ×100). ChMC had longer, spindly processes in comparison to the other two cell types.

AMD is a leading cause of vision loss in elderly, and its pathogenesis is significantly impacted by various inflammatory processes [31,32]. Although immunomodulatory roles have been recently attributed to choroidal melanocytes (ChMC) [22,33], little is known about their contribution to pathophysiology of dry and wet AMD. The ability to culture ChMC has been instrumental in advancing our knowledge regarding their biology and potential functions. Using wild-type Immortomice, we isolated and characterized choroidal melanocytes (ChMCs). The cell pellet during the final centrifugation in isolation was pigmented, indicating the cells we obtained contained melanin. Figure 1 shows the morphology of ChMC shortly after isolation and through passage 3. Many cells in the earliest stages of growth (P0) contained some level of pigmentation. However, fewer cells contained pigmentation with each successive passage until very few pigmented cells were observed by the third passage, as previously noted with human ChMC [7]. We compared the morphology of later passage cells (P7) to other cell types isolated from choroidal tissue to show that the cells isolated here were a unique cell type. Figure 2 shows the morphology photos of ChMC, ChEC, and RPE cells. The ChMC isolated here often had two or three long, thin processes and grew in a somewhat disorganized manner on the surface of the culture plate. The ChEC also had long processes but tended to grow in a mesh-like network on the culture plate surface. RPE cells have slightly shorter processes than both of the other cell types and are organized in a cobblestone pattern with tighter junctions between cells than the ChMC.

**Figure 3:**
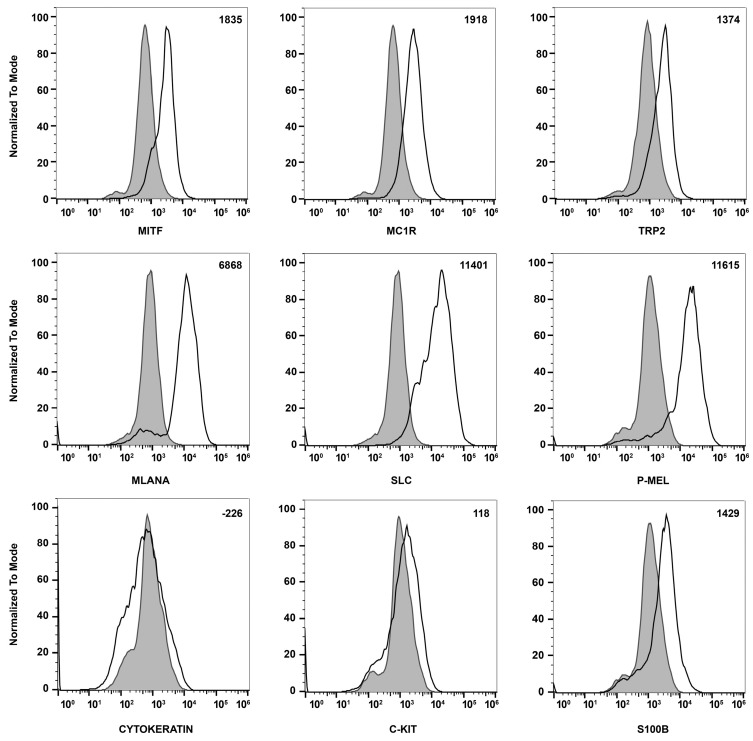
Expression of melanocyte markers in ChMC. Mouse ChMC were examined for expression of Mitf, Mc1r, Trp2, Mlana, Slc, P-Mel, Pan Cytokeratin (RPE marker), c-Kit, and S100β using flow cytometry. The shaded areas show staining in the absence of primary antibody (secondary control), and the unshaded peaks show staining with primary antibody. The difference between the geometric means of primary antibody-stained cells and secondary antibody-stained control cells can be found in the top right corner of each graph. These experiments were performed at least 2 times with 3 different isolations of ChMC with similar results.

**Figure 4:**
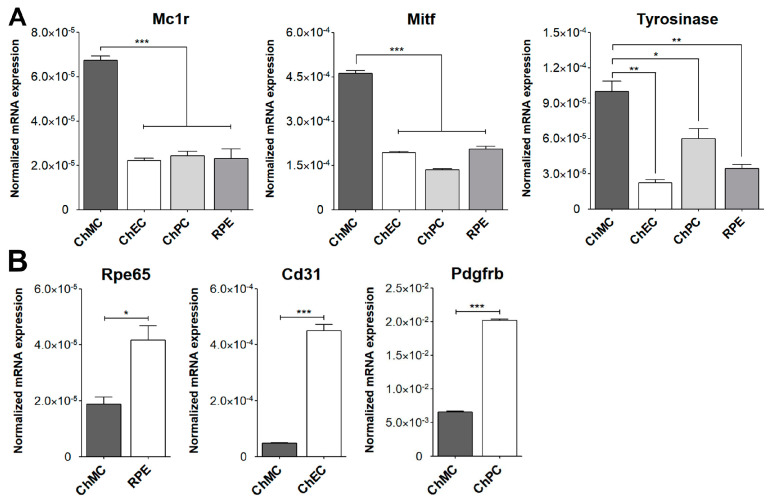
Expression of ChMC, ChEC, ChPC, and RPE cell-specific markers. RNA was prepared from various cell types, and the expression level of desired markers were assessed using RT-qPCR. (**A**) Expression of melanocyte markers and (**B**) expression of other cell-specific markers including Rpe65 (RPE cells), Cd31 (ChEC), and Pdgfrb (ChPC). Please note the specific expression of melanocyte markers in ChMC, while the expression of other cell type markers was negligible in ChMC. These experiments were repeated with three isolations of each cell type with similar results. (*** p < 0.001, ** p < 0.01, and * p < 0.05; n = 3; (**A**) one-way ANOVA with Tukey’s multiple-comparison test, (**B**) two-tailed *t*-test).

To confirm that these cells were melanocytes, we examined the expression of the melanocyte and melanin-producing cell markers Mitf, Mc1r, Trp2, Mlana, Slc, Pmel, c-Kit, and S100β using flow cytometry analysis. We also examined the expression of cytokeratin, an RPE cell marker, both by FACS and immune staining, to determine if these cells are comparable with ChMC. Figure 3 shows that the cells isolated here express the melanocyte markers, but not the RPE (cytokeratins) cell marker, further confirming their identity as melanocytes. We also compared the expression of ChMC specific markers by qPCR in ChMC, ChEC, ChPC, and RPE cells. Figure 4A shows that Tyrosinase, Mc1r, and Mitf are predominantly expressed in ChMC. Figure 4B shows the expression of RPE (Rpe65)-, ChEC (Cd31)-, and ChPC (Pdgfrb)-specific markers compared to ChMC. As expected, ChMC showed a negligible expression of these markers.

**Figure 5:**
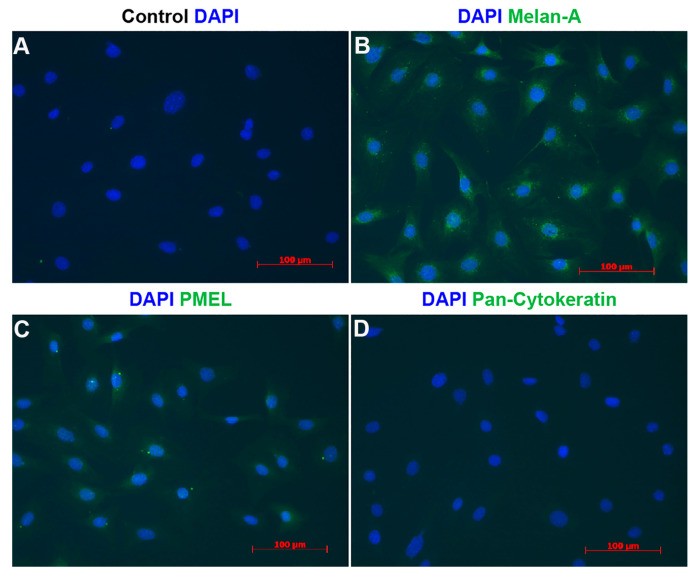
Indirect immunofluorescence staining of melanocytes. Cells were prepared and stained using DAPI (**A**), Melan-A (**B**), Pmel (**C**), and pan-Cytokeratin (**D**) antibodies as detailed in Section 2. DAPI was used to stain nuclei of the cells. Scale bars = 100 μm. Please note specific staining of the majority of cells with Melan-A (**B**) and Pmel (**C**), and lack of staining for pan-Cytokeratin (**D**). This experiment was repeated with 3 isolations of ChMC with similar results.

To further determine the purity of ChMC cultures established here, we immunostained cells for melanocyte and RPE markers. Figure 5 demonstrates that these cells express the melanocyte markers (Pmel and Melan A) but not the RPE marker (Pan Cytokeratin). Nearly all cells were positive for melanocyte markers and lacked the expression of cytokeratins as expected for melanocytes. Collectively these data strongly support the identity of the ChMC prepared here.

### 3.2. Expression of Cell Adhesion Proteins in ChMC

**Figure 6:**
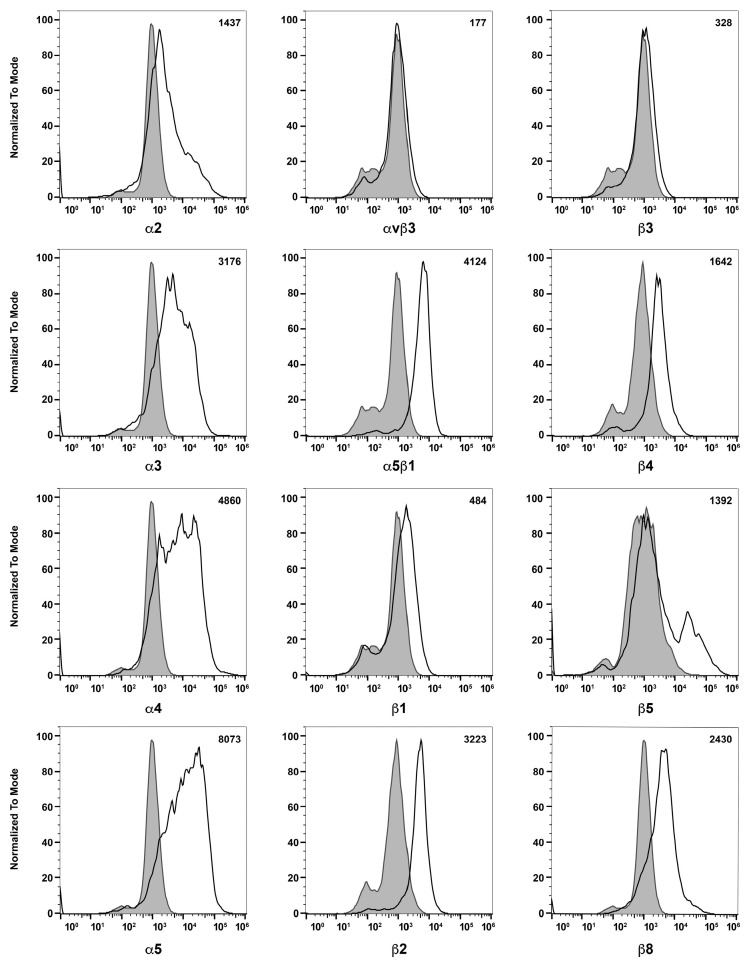
Expression of integrins in choroidal melanocytes in culture. The expression of α2, α3, α4, α5, αVβ3, α5β1, β1, β2, β3, β4, β5, and β8 integrins was determined using flow cytometry, as described in Section 2. The shaded areas show staining in the absence of primary antibody (secondary control), and the unshaded areas show staining with primary antibody. The difference between the geometric means of primary antibody-stained cells and secondary antibody control-stained cells can be found in the upper right corner of each graph. These experiments were repeated with two isolations of ChMC with similar results. Please note that α and β integrins were expressed at significant levels with the exception of β3 and αvβ3 integrins.

**Figure 7:**
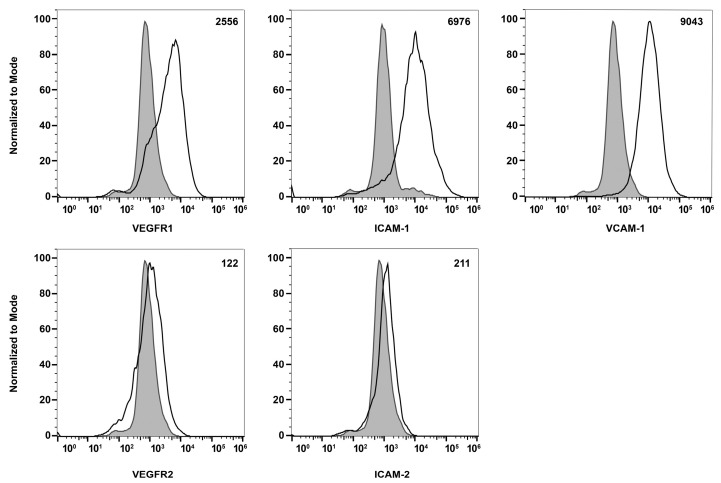
Expression of other cell adhesion and growth factor receptors. The expressions of VEGF-R1, VEGF-R2, ICAM-1, ICAM-2, and VCAM-1 were determined using flow cytometry, as described in Section 2. The shaded areas show staining in the absence of primary antibody (secondary control), and the unshaded areas show staining with specific primary antibody. The difference between the geometric means of primary antibody-stained cells and secondary antibody control-stained cells can be found in the upper right corner of each graph. These experiments were repeated with two isolations of ChMC with similar results.

To learn about melanocytes’ cell–cell and cell–matrix interactions, we examined the expression of various cell adhesion molecules on their surface by flow cytometry. First, we tested if these ChMC express integrins α2, α3, α4, α5, αVβ3, α5β1, β1, β2, β3, β4, β5, and β8. Figure 6 shows that ChMC moderately express (geometric mean shifts between 500 and 2000) α2, β4, and β5, and strongly express (geometric mean shifts over 2000) α3, α4, α5, α5β1, β2, and β8. Integrins αvβ3, β1, and β3 are not well expressed (geometric mean shifts under 500) in these cells. We also examined other cell adhesion markers by flow cytometry, specifically VEGF-R1, VEGF-R2, ICAM-1, ICAM-2, and VCAM-1. Figure 7 shows that our ChMC strongly expressed (geometric means over 2000) VEGF-R1, ICAM-1, and VCAM-1, but negligibly expressed (geometric means under 300) VEGF-R2 and ICAM-2, more specific EC markers.

### 3.3. Localization of Melanocytes in the Choroid

**Figure 8:**
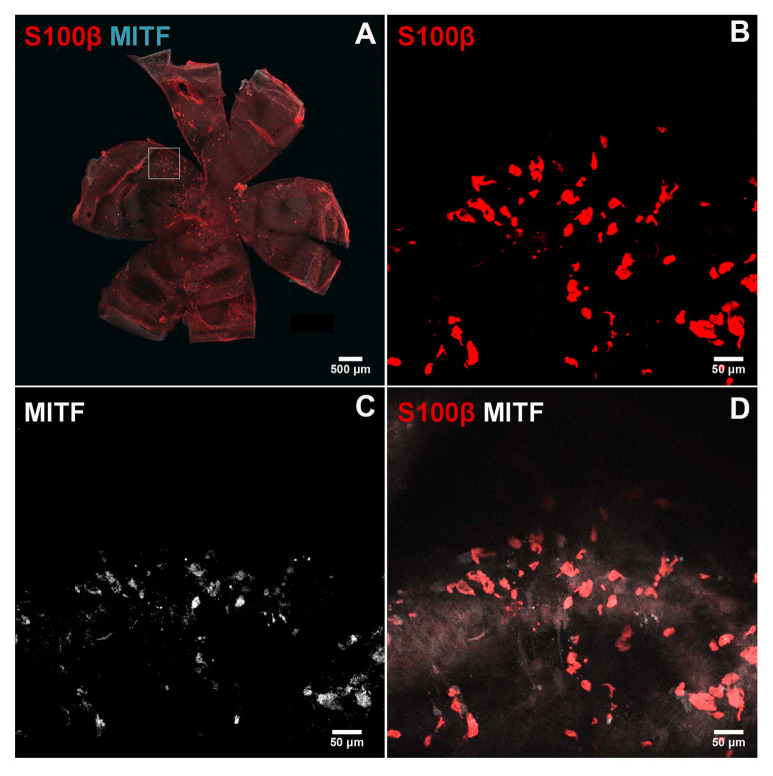
Immunostaining of the melanocytes in the choroidoscleral complex. Choroid/RPE wholemounts were prepared from 8-week-old FVB/NJ mice and stained with specific antibodies as detailed in the Section 2. Melanocytes positive for S100β ((**B**); red) and MITF ((**C**), cyan or white) were detected in the choroidoscleral complex. A low magnification of wholemount double staining is shown in (**A**). Higher magnification of double staining is shown in (**D**). Images were captured using Nikon A1 confocal microscope and processed using NIS-Elements Software. Scale bar = 50 µm. These experiments were repeated with eyes from at least 5 mice with similar results.

We recently reported a successful method of staining mouse RPE/choroid tissues from pigmented and albino mice for the evaluation of choroidal vasculature, mast cells, and macrophages [34]. Here, using this method, we evaluated the distribution of melanocytes in the choroid of 8-week-old albino (FVB/NJ) mice using two specific markers of melanocytes, namely Mitf and S100β (Figure 8). Panel A shows RPE/choroid wholemount at low magnification stained with both antibodies. Panel B shows staining for S100β (red), Panel C shows staining for Mitf (white), and Panel D shows the overlay. Thus, a large number of melanocytes are present in the choroid that express melanocyte markers.

### 3.4. Melanogenic Responses of Mouse ChMC

**Figure 9:**
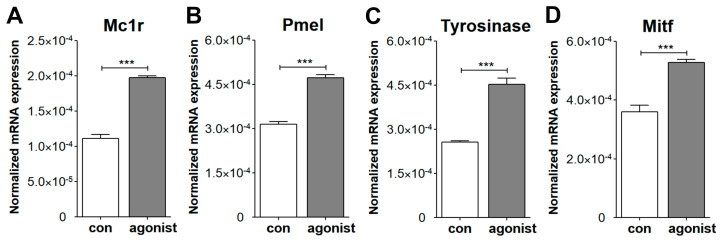
Evaluation of ChMC for melanogenesis through activation of Mc1r. ChMC were incubated with Mc1r agonist BMS 470,539 for 72 h. Following incubation, total RNA was prepared and subjected to RT-qPCR analysis using specific primers (Table 1) for melanogenic genes including Mc1r (**A**), Pmel (**B**), tyrosinase (**C**), and Mitf (**D**) as detailed in Section 2. Please note significant up-regulation of these genes in ChMC incubated with the Mc1r agonist compared with control (vehicle). These experiments were repeated with two isolations of ChMC. (*** p < 0.001; n = 3; two-tailed *t*-test).

During the isolation of ChMC, we noted progressive loss of pigment (Figure 1), as previously reported with human ChMC cultures [7]. We next asked whether ChMC retained the capacity to respond to a melanogenic stimulus. Melanocyte-stimulating hormone (α-MSH) stimulates melanogenesis in skin melanocytes in response to UV radiation through melanocortin receptor 1 (MC1R) [35,36]. We showed that mouse ChMC expresses Mc1r (Figure 3). Here, we asked whether the stimulation of Mc1r stimulates melanogenesis in mouse ChMC. ChMCs were incubated with Mc1r agonist, BMS 470,539 (1 µM), for 72 h, and RNA was isolated for gene expression studies by qPCR. We examined the expression of Mcr1, Pmel, Tyrosinase, and Mitf, genes with known action in melanogenesis [37]. We noted a significant increase in the expression of these genes in ChMC incubated with Mc1r agonist (Figure 9). Thus, ChMCs have the capacity to undergo melanogenesis in response to the activation of Mc1r, and perhaps other stressors, such as UV exposure, as reported for skin melanocytes.

### 3.5. Expression of Stem Cell Factor (Scf) and Its Receptor (c-Kit) in ChMC

**Figure 10:**
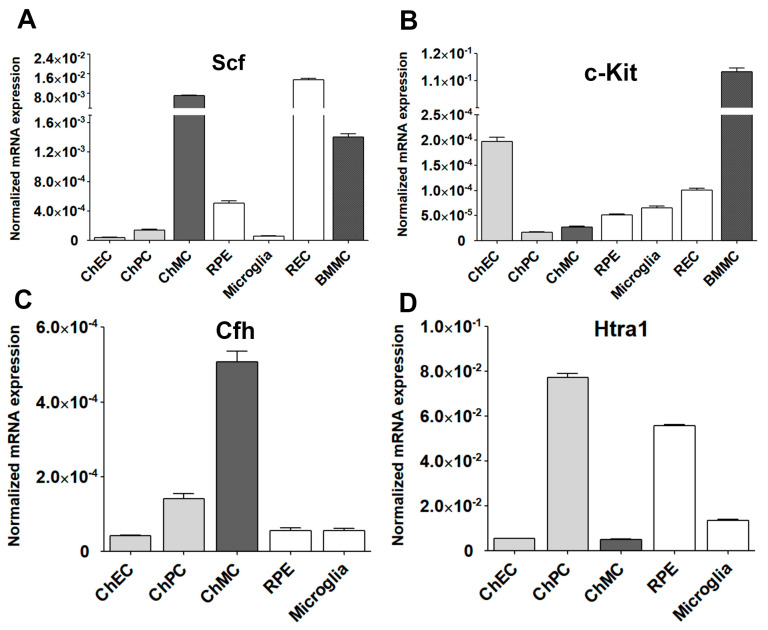
Expression of Stem cell factor (Scf) and its receptor (c-Kit) in ChMC. Total RNA was isolated from actively growing ChMC, as well as ChEC, ChPC, RPE, Microglia, REC (retinal EC), and BMMC (bone-marrow-derived mast cells). (**A**) The expression of Scf and (**B**) c-Kit was determined by RT-qPCR analysis using specific primers (Table 1). Please note the significant expression of Scf and lower expression of c-Kit in ChMC compared to the other cell types examined. This experiment was repeated with two different isolations of these cells. We also similarly examined the expression of Cfh (complement factor h; (**C**)) and Htra1 (high-temperature requirement a1; (**D**) in these cells. Please note significant expression of Cfh and lower expression of Htra1 in ChMC compared to other cell types examined here.

The tyrosine kinase receptor (c-Kit) and its ligand stem cell factor (Scf) interactions mediate a wide range of biological activities including the differentiation and proliferation of melanocytes [38,39,40]. Altered proliferation of choroidal melanocytes could result in rapid vision loss. c-Kit is expressed in ChMC and its stimulation with Scf in cultured human ChMC could stimulate proliferation without impacting cell morphology or melanin production [21]. We examined the expression of c-Kit and its ligand (Scf) in mouse ChMC as well as other cell types (Figure 10A,B). ChMC expressed lower levels of c-Kit compared to other cell types examined except ChPC. However, ChMC expressed higher levels of Scf compared to the other cells examined and was comparable to REC. Thus, a high level of Scf produced by ChMC could act in paracrine to modulate the activity of cells expressing high levels of its receptor c-Kit including ChEC and likely choroidal mast cells, the key innate immune cells in the choroid.

We also examined the expression of two genes with polymorphisms that are highly associated with the pathophysiology of AMD, namely complement factor H (Cfh) and High-temperature requirement a1 (Htra1) [41,42] (Figure 10C,D). ChMC showed the highest Cfh expression compared to other cell types including RPE cells, which are reported as a major source of Cfh [43]. In contrast, ChMC expressed lower levels of Htra1 compared to RPE cells, while ChPC expressed the highest levels of Htra1. Thus, the level of Cfh expression in ChMC is consistent with their proposed role in regulating inflammatory processes.

### 3.6. Inflammatory Responses of ChMC to Various Extracellular Stressors

**Figure 11:**
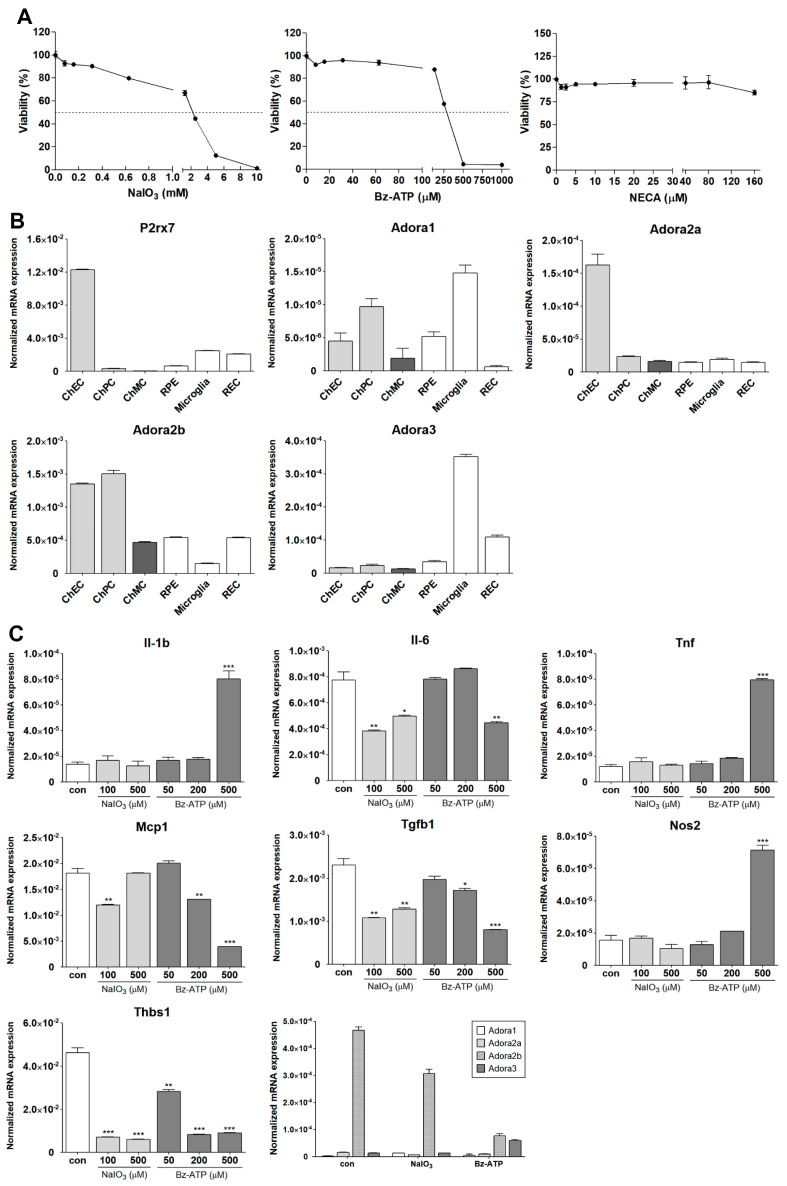

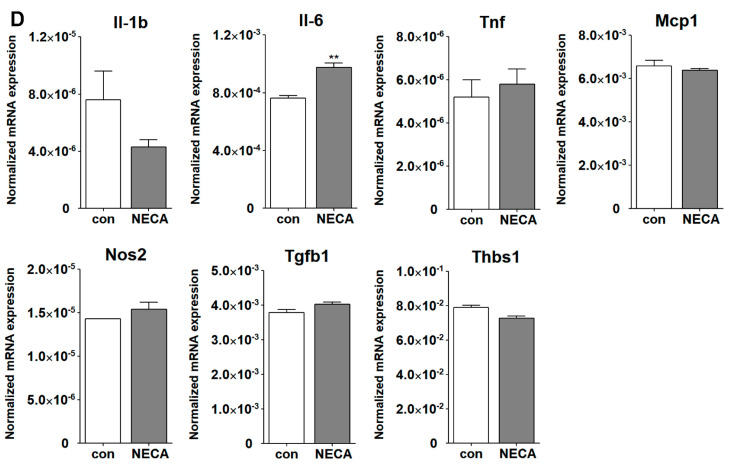
Inflammatory responses of ChMC to various stressors. (**A**) Dose response curve for ChMC incubated with various concentrations of NaIO_3_, Bz-ATP (ATP analog), and NECA (adenosine analog) for 24 h. Cell viability was assessed as detailed in Section 2. The dashed line shows the corresponding 50% viability dose. Please note sensitivity of ChMC to NaIO_3_ and Bz-ATP and lack thereof with adenosine. (**B**) Expression of ATP receptor (P2rx7), and adenosine receptors (Adora1, Adora2a, Adora2b, and Adora3) were examined by RT-qPCR using RNA from indicated cell types and specific primers (Table 1). Please note the expression of P2rx7 was lower in ChMC compared to other cell types, with the highest in ChEC. ChMC expressed lower levels of Adora1, Adora2a, and Adora3, but expressed relatively higher levels of Adora2b. Adora1 was the highest in microglia and ChPC. ChEC expressed the highest level of Adora2a, while Adora2b was higher in ChEC and ChPC, and Adora3 was highest in microglia followed by REC. (**C**) Expression of inflammatory mediators in ChMC incubated with NaIO_3_ (0, 100, and 500 µM) and Bz-ATP (0, 50, 200, and 500 µM) for 24 and assessed by RT-qPCR analysis using specific primer. Il-1b, Tnf, and Nos2 level only increased with 500 µM Bz-ATP, with NaIO_3_ having no effect. Il-6, Mcp1, Tgfb1, and Thbs1 levels decreased by both NaIO_3_ and Bz-ATP. NaIO_3_ did not increase the expression of any of the examined inflammatory mediators. Both NaIO_3_ and Bz-ATP reduced Adora2b, but only Bz-ATP stimulated Adora3 expression in ChMC. (**D**) Expression of inflammatory mediators in ChMC incubated with NECA 100 µM for 24 h. RNA was isolated and used for RT-qPCR analysis of gene expression using specific primers (Table 1). NECA only increased IL-6 expression without a significant effect on the expression of other mediators examined here. Con (Vehicle control). (*** p < 0.001, ** p < 0.01, * p < 0.05; n = 3; (**C**) one-way ANOVA with Tukey’s multiple-comparison test, (**D**) two-tailed *t*-test).

ChMC are proposed to be involved in the regulation of choroidal inflammatory homeostasis [22,29,30]. A recent RNA sequencing study demonstrated that lipopolysaccharide (LPS) stimulation of human ChMC resulted in a proinflammatory transcription state along with gene expression patterns impacting cellular adhesive and migratory properties [30]. However, the direct role of these gene expression changes in melanocytes, and their role in the pathophysiology of AMD, remains to be further explored. The ability to culture and study ChMC isolated from wild-type and transgenic mice will have a significant impact on such studies. Here, we assessed whether stressors that impact ocular oxidative stress and inflammatory processes, including NaIO_3_, ATP, and adenosine, affect ChMC inflammatory activity. First, we evaluated the sensitivity of ChMC to these stressors by examining the viability of ChMC incubated with various stressor concentrations. We used Bz-ATP (an ATP analog) and NECA (an adenosine analog) for these studies. Figure 11A shows the viability of ChMC incubated with various concentrations of NaIO_3_, Bz-ATP, and NECA. Although ChMC showed minimal sensitivity to various NECA concentrations, they exhibited sensitivity to NaIO_3_ and Bz-ATP, with IC_50_ values of 2 mM and 250 µM, respectively.

Receptors for ATP and its metabolites (purinergic receptors) can mediate stress and inflammatory processes [44,45]. We next examined the expression of receptors for ATP (P2rx7) and adenosine (Adora1, Adora2a, Aadroa2b, and Adora3). Figure 11B shows the expression of P2rx7 and various adenosine receptors in ChMC as well as other choroidal cells including ChEC, ChPC, RPE, microglia, and REC. ChMC expressed lower levels of the P2rx7 receptor compared to other cell types, but they expressed comparable levels of adenosine receptors when compared to other cell types examined.

We next determined the impact of various extracellular stressors on the inflammatory characteristics of ChMC. Figure 11C shows the expression of inflammatory factors including Il-1β, Il-6, Tnf-α, Mcp-1, Tgf-β1, Nos2, and Thbs1 in response to treatment with two doses of NaIO_3_ (0.1 and 0.5 mM) and three concentrations of Bz-ATP (50, 200, and 500 µM). The expression of IL-1β, Tnf-α, and Nos2 was significantly upregulated by incubation with Bz-ATP, while the expression of Il-6, Mcp-1, Tgf-β1, and Thbs1 was downregulated. NaIO_3_ treatment did not result in an increased expression of any of the genes examined. However, the expression of Il-6, Tgf-β1, and Thbs1 was decreased with NaIO_3_ treatment. We also examined the expression of adenosine receptors in cells incubated with NaIO_3_ or Bz-ATP. The expression of Adora2b was downregulated by NaIO_3_ and Bz-ATP, while only Bz-ATP increased the expression of Adora3. The expression of other adenosine receptors was not affected. Figure 11D shows that NECA treatment minimally affected the expression of the inflammatory mediators examined, with the exception of Il-6, of which its expression increased with NECA treatment.

## 4. Discussion

Choroidal melanocytes (ChMCs) contribute to the morphogenesis and maintenance of the choroidal vasculature and are normally abundant around well-developed blood vessels. Mice deficient in melanocytes (Mitf-M mutant) have a significantly thinner choroid with an irregular choroidal vasculature [1]. However, the underlying mechanisms involved remain unknown and benefit from further elucidation of melanocyte development and function. Although ChMC isolation from human eyes has been previously reported, to the best of our knowledge, ChMC isolation from mouse eyes has not been previously described. Here, using a novel strategy that we have previously used to isolate various other cell types from mouse eyes and other tissues, we report successful isolation of murine ChMC.

We extensively characterized the murine ChMC and showed that they express most of the known cell type specific marks for melanocytes, but not those for ChEC, ChPC, or RPE cells. We also show ChMC express various integrins, cell adhesion markers, and growth factor receptors on their cell surface. ChMC also constitutively expressed high levels of VCAM-1 and ICAM-1 on their surface that could facilitate their interaction with proinflammatory cells including monocytes. Unsurprisingly, ChMC showed very low levels of VEGF-R2 and ICAM-2, which are more specific markers of ChEC. Using our novel immunostaining of RPE/choroid wholemount in mice [34], we showed that these cells are specifically labeled in the choroid using antibodies to melanocyte markers.

The murine ChMC also expressed significant amounts of Scf of which interaction with its receptor c-Kit impacts recruitment and choroidal cell characteristics [46]. Mice deficient in mast cells (c-Kit mutant) exhibit some of the characteristics of melanocytes-deficient mice (Mitf-M mutant) such as white coat color and black eyes. ChMC also expressed c-Kit and its ligand Scf as demonstrated here. Thus, unraveling the inter-relationship between the signaling pathways mediated by c-Kit and Mitf-M will aid in a better understanding of the role these cells play in choroidal development and function and how their coordinated alterations may contribute to inflammatory processes and pathogenesis of AMD.

Melanocortin receptor 1 (MC1R) mediates melanin production in the skin melanocytes [47], but its role in ChMC and melanin production has been debated. Human ChMCs were initially shown to lack MC1R expression and fail to undergo melanogenesis [48]. However, recent studies using more sensitive techniques have confirmed the expression of MC1R in human ChMC and their response to α-MSH by the upregulation of melanogenic gene expression [30]. This is also consistent with the ability of ChMC to retain the capacity to produce pigments throughout life, which is not seen in RPE cells [49]. The murine ChMC prepared here also expressed Mc1r, of which activation by a specific agonist enhanced the expression of key genes involved in melanogenesis, including *Pmel*, *Tyrosinase*, and *Mitf*. Furthermore, the expression of *Mc1r* in ChMC was also increased with agonist treatment. Thus, murine ChMC expresses Mc1r which could respond to α-MSH stimulation-enhancing melanogenesis as occurring in dermal melanocytes upon UV exposure. However, whether this occurs in the choroid in vivo needs further verification.

The role of melanocytes in choroidal homeostasis and the regulation of inflammatory processes, especially with aging, and its contributions to AMD pathogenesis, remain a great interest in the field. The incubation of ChMC with TNF-α and INF-ɣ promotes the release of MCP1/CCL2- and ICAM-1-stimulating monocyte migration [4]. In addition, co-culturing ChMC with activated T cells blocked their proliferation, suggesting ChMC plays a significant role in maintaining the immune privilege of the eye. These results indicate that ChMCs are immunologically competent and can constitutively express and release various inflammatory mediators, which can be further induced by systemic inflammatory mediators. Thus, ChMCs are likely to contribute to eye diseases with an inflammatory component including uveal melanoma, AMD, and uveitis.

NaIO_3_ has been extensively used to mediate outer retinal degeneration in various preclinical models for studies of dry AMD pathogenesis with RPE cells as a key target [50,51,52]. RPE cells regulate choroidal development, and, in the absence of RPE, choroid fails to develop [53]. However, the contribution of ChMC to these activities and the impact NaIO_3_ and other stressors have on ChMC characteristics remain largely unknown. A recent study showed that Indian hedgehog (Ihh; produced by ChEC) signaling though Gli1^+^ stromal cells (ChPC) enhances the proliferation and survival of ChMC and mast cells. The absence of this signaling resulted in enhanced inflammatory responses in NaIO_3_-mediated RPE/choroid injury [29]. Here, we began to address how the incubation of ChMC with NaIO_3_, and other potential stressors and mediators of inflammatory processes including extracellular ATP [44,54] and its metabolite adenosine [55,56], impact ChMC inflammatory responses. This was addressed by examining the expression of inflammatory mediators that have been previously shown to be expressed by human ChMC. Our viability studies indicated that murine ChMC tolerated relatively high concentrations of adenosine analog NECA but showed sensitivity to NaIO_3_ and ATP analog Bz-ATP treatments.

Here, we showed that the receptors mediating ATP (P2rx7) and adenosine (Adora1, Adora2a, Adora2b, and Adora3) activities are expressed in ChMC. We also found that NaIO_3_ and Bz-ATP downregulated P2rx7 (a key ATP receptor) expression in ChMC. Adora2b was the predominant receptor expressed in ChMC, with other adenosine receptors expressed at lower levels. Although the impact of NaIO_3_ and Bz-ATP on Adora1 and Adora2a adenosine receptors was minimal, Bz-ATP significantly increased the Adora3 but decreased Adora2b expression. However, the significance of these changes in the regulation of ChMC inflammatory activities requires further investigation.

We next assessed the impact of these stressors on expression of various inflammatory mediators in ChMC. We found that the incubation of ChMC with NaIO_3_ minimally impacted the expression of Il-1β, Tnf-α, and Nos2. However, NaIO_3_ treatment resulted in decreased expression of Il-6, Mcp-1, Tgf-β1, and Thbs1. Il-6 and Mcp-1 are generally referred to as proinflammatory cytokines. However, Tgf-β1 and Thbs1 generally have anti-inflammatory activities. Thus, Tgf-β1 and Thbs1 downregulation could support a proinflammatory choroidal environment [57,58]. NaIO_3_ did not increase the expression of any of the inflammatory mediators examined here. However, the overall changes noted could promote a proinflammatory environment in the choroid.

The incubation of ChMC with Bz-ATP decreased the expression of IL-6, Mcp-1, Tgf-β1, and Thbs1. In contrast, Bz-ATP increased the expression of Il-1β, Tnf-α, and Nos2. Although the expression changes for Il-6, Mcp-1, Tgf-β1, and Thbs1 were similar under both treatments, the increase in IL-1β, Tnf-α, and Nos2 expression was only noted in ChMC incubated with Bz-ATP compared to NaIO_3_. The differences could be related to the engagement of different signaling pathways, which require further elucidation. The molecular and cellular targets engaged by NaIO_3_ treatment remain unknown, and its impact on outer retinal degeneration is merely linked to increased oxidative stress, leading to the death of RPE cells followed by loss photoreceptor cells [59,60]. However, we propose that other shared mechanisms of action could be linked to the release of extracellular ATP in response to NaIO_3_ stress and similar inflammatory impacts as seen with Bz-ATP. The incubation of ChMC with NECA minimally impacted the expression of inflammatory mediators examined, including Il-1β, Tnf-α, Mcp-1, Nos2, Tgf-β1, and Thbs1, while Il-6 expression was increased. These results are consistent with the minimal cytotoxicity noted in cells incubated with high NECA concentrations.

Some of the limitations of the studies presented here include the examination of protein levels for genes with expressions that were determined at mRNA levels, including many inflammatory agents and purinergic receptors. The production of many inflammatory mediators examined here by qPCR were previously reported in human ChMC at protein levels. Here, we confirmed their expression in murine ChMC. However, the changes noted in mRNA levels in murine ChMC need to be verified in future experiments by immunostaining and Western blot analysis. The other limitation of our studies is that the morphology comparisons, among different choroidal cells, were qualitative, and more quantitative assessments may provide unique differences among the choroidal cells. Nonetheless, we demonstrated that the murine ChMC isolated here exhibits the majority of properties previously demonstrated for human ChMC and expresses the key melanocyte markers. We also provide additional characteristics of murine ChMC cell adhesive molecules, which are important in the modulation of their cell–cell and cell–matrix interactions.

## 5. Conclusions

In summary, here, we present a method for the isolation of murine ChMC. ChMC as a predominant cell type in the choroid is vital to the development, homeostasis, and function of the choroid. In addition, its role in the modulation of inflammatory processes in the choroid is crucial to the preservation of vision. We showed that murine ChMCs, like human ChMCs, express major melanocyte markers, as well as many inflammatory mediators. The delineation of the regulatory mechanisms that impact the interaction of ChMC with other choroidal and inflammatory cells and the regulation of their inflammatory characteristics will provide novel targets for the restoration of choroidal function and treatment of sight threatening eye diseases such as dry AMD.

## Figures and Tables

**Table 1:** The PCR primer sequences.

Genes	Forward 5′ to 3′	Reverse 5′ to 3′
Adora1	GTCAAGATCCCTCTCCGGTA	CAAGGGAGAGAATCCAGCAG
Adora2a	GGTCCTCACGCAGAGTTCC	TCACCAAGCCATTGTACCG
Adora2b	CCGATATCTGGCCATTCG	AGTCAATCCAATGCCAAAGG
Adora3	CTCTTTGCTAGGATTGCTTGG	AGAAGGAATGCCAAGAGCAG
Cd31	GTGTGGAAGCCAACAGCCA	TCCATTAAGGGAGCCTTCCG
Cfh	GGGCAAGTGGAAGTGATGTG	TGTCAATAGGTGGAGGAGGC
c-kit	GAAGGACTCCTCCTGCTTTAGA	CAAATACACTCAGGGGAGCAC
Htra1	GGATGTGGATGAAAAGGCGG	AATTCTCCAGGTCTCAGCTCTG
Il-1b	GTTCCCATTAGACAACTGCACT	CCGACAGCACGAGGCTTTT
Il-6	CAACCACGGCCTTCCCTACT	TTGGGAGTGGTATCCTCTGTGA
Mc1r	CTACAAGCACACAGCCGTTC	AGTGCCAGCATGGCTAGAAA
Mcp1	GTCTGTGCTGACCCCAAGAAG	TGGTTCCGATCCAGGTTTTTA
Mitf	GGTGACAACATAGGGAATGGTT	CCAGTCCCTGAAGAATCCA
Nos2	GGCAGCCTGTGAGACCTTTG	CATTGGAAGTGAAGCGTTTCG
P2rx7	CGTCTTTTCCTACATTAGCTTTGC	ATGCCTTTGACCTTGGTGTG
Pdgfrb	ATCGCGCCACCTTAATCAAC	GCTAAGAAGTCCATGCCGTT
Pmel	CTCTTGTTTCCTGTGGTTCCT	GTAGTGGTTCCTTGCCTAGATG
Rpe65	CCTGGTTCTGAATGCCAAAG	CATGGAAGGTCACAGGGATATT
SCF	AATGAATGGAAAAATCTGTTGTGTAA	TGCGTACAGAATAGCTAAGATTTCA
Tgfb1	GCAGTGGCTGAACCAAGGA	AGCAGTGAGCGCTGAATCG
Thbs1	TGGCCAGCGTTGCCA	TCTGCAGCACCCCCTGAA
Tnf	ACCGTCAGCCGATTTGCTAT	TTGACGGCAGAGAGGAGGTT
Tyrosinase	GGGATTGGAGAGATGCAGAAA	TCTGCCAGGAGGAGAAGAA

## Data Availability

All the data supporting reported results are included in the manuscript.
